# Supportive treatment of vascular dysfunction in pediatric subjects with obesity: the OBELIX study

**DOI:** 10.1038/s41387-021-00180-1

**Published:** 2022-01-10

**Authors:** Luca Pecoraro, Thomas Zoller, Richard L. Atkinson, Fulvio Nisi, Franco Antoniazzi, Paolo Cavarzere, Giorgio Piacentini, Angelo Pietrobelli

**Affiliations:** 1 Department of Medicine, University of Verona, Verona, Italy; 2 Paediatric Clinic, ASST Mantua, Mantua, Italy; 3 Pediatric Unit, Department of Surgical Sciences, Dentistry, Gynecology and Pediatrics, University of Verona, Verona, Italy; 4 Department of Medicine, Virginia Commonwealth University, Richmond, VA USA; 5 Humanitas Clinical and Research Center—IRCCS, Rozzano, MI Italy; 6 Pennington Biomedical Research Center, Baton Rouge, LA USA

**Keywords:** Cardiovascular diseases, Obesity

## Abstract

**Introduction:**

Overweight or obese children develop abnormal endothelial cell dysfunction and arterial intima–media thickening with increased vasomotor tone and inflammation. *Curcumin*, *resveratrol*, zinc, magnesium, selenium, and vitamin D have shown beneficial effects on endothelial function. We test, among overweight and obese pediatric subjects, the effects on the endothelium of a combination of *curcumin*, *resveratrol*, zinc, magnesium, selenium, and vitamin D.

**Methods:**

Forty-eight subjects (6–17 years) were randomized into two groups (placebo vs treatment) attended three visits at 0, 3, and 6 months (±15 days). Endothelial function was assessed by means of a post-occlusive release hyperemic (PORH) test for estimation of delta flow (DF) and hyperemic AUC index, and a heat provocation test (HPT) to measure DF HPT (DF_HPT_).

**Results:**

Significant DF difference was noted at 6 months in both groups (*p* < 0.001). Overall time trend was significantly different between baseline, 3 months, and 6 months both in placebo (*p* < 0.05) and treatment (*p* < 0.001) groups and their comparison (*p* < 0.001). No differences were noted in hyperemic AUC index (3 and 6 months), whilst there were significant differences in time trends of rreatment (*p* < 0.001) and placebo (*p* < 0.05) groups and their comparison (*p* < 0.001). DF_HPT_ difference between groups was significant at 3 and 6 months (*p* < 0.05). The overall time trend was significant exclusively in Treatment group between 3 and 6 months (*p* < 0.05). Correlation with anthropometrics was found for DF and body mass index (*r* = 0.677 6 months, *p* < 0.05), as well as for hyperemic AUC index and males (*r* = 0.348, *p* < 0.05), while DF_HPT_ showed no correlation.

**Conclusion:**

C*urcumin*, *resveratrol*, zinc, magnesium, selenium, and vitamin D appear to be promising in enhancing endothelial function by improvement of both DF in the PORH test and DF in the HPT, lowering the risk of developing cardiovascular diseases in overweight and obese pediatric subjects.

## Introduction

Pediatric overweight and obesity are traditionally characterized by an excess of body fat [1], which is an independent cardiovascular risk factor that could lead to type 2 diabetes, hypertension, insulin resistance, and reduced endothelial function development [2]. The link between excess body fat, endothelial dysfunction, and insulin resistance is related to the fact that endothelium-dependent vasodilatation is impaired in proportion to insulin resistance and other adiposity-related indices [3].

Bussey and colleagues in 2016 found reduced nitric oxide (NO) production and increased inflammation in perivascular adipose tissue (PVAT) of obese mice compared to nonobese controls. The same study found significantly improved PVAT anticontractile function after weight loss by reduced adipose inflammation and increased NOS availability [4].

Similar results were previously stated by Ketonen et al. in 2010, they found impaired endothelium-dependent vasodilation in response to acetylcholine in obese mice receiving high-fat diet in comparison with mice receiving a normal fat diet. Differences between the obese and control groups markedly reduced after the introduction of caloric restriction in the obese group [5].

Moreover, it is very well known that adipose tissue is a key regulator of inflammation with the secretion of proinflammatory cytokines (i.e., adipokines) that play a role in influencing glucose metabolism and endothelial function [6]. Children who are overweight or obese develop abnormal endothelial cell dysfunction and arterial intima–media thickening with increased vasomotor tone and inflammation [7, 8]. This may lead to atherosclerotic plaques formation [9]. The endothelium contributes to blood pressure and flow regulation by releasing nitric oxide (NO) and other compounds, which contribute either to vasodilation or vasoconstriction [10, 11]. On the other hand, the interaction between endothelium and adipokines suggests a role for adipokines in vascular homeostasis and ultimately in the mechanisms for the development of cardiovascular diseases [12–14]. Also, a healthy endothelium prevents platelet aggregation, proliferation of vascular smooth muscle cells, adhesion, and subsequent diapedesis of leukocytes through the vascular wall [15]. The endothelium plays a unique role in vascular homeostasis that is maintained by endothelium-derived biomolecules with different functions (i.e., vasodilation, vasoconstriction, growth promoter, growth inhibitor, adhesion molecules, thrombolytic factors) [15]. Endothelium-dependent damage arises from metabolic abnormalities of glucose metabolism that lead to vascular dysfunction [15]. Endothelial dysfunction characterized by abnormal vasodilator response and increased arterial stiffness is associated with an increased risk of cardiovascular events [15] and is present in pediatric subjects with obesity [11]. The major goal of obesity therapy in children should be the reduction of the long-term risks of cardiovascular diseases. Since damaged endothelium is so involved in the development of later risks of morbidity and mortality, it may be useful to monitor and eventually treat endothelium status in order to prevent long-term risk factors [16–20]. Among different therapies, *curcumin* showed promising results in inhibition of advanced glycation end product-induced oxidative stress and inflammatory responses in endothelial cell damage [14]. *Curcumin* is the most active component of the curcuminoids extracted from *Curcuma longa* L., and it has been demonstrated to protect against cellular inflammatory responses and oxidative stress in vascular complications as well as endothelial damage [16, 17]. Another antioxidant, *resveratrol*, has shown beneficial effects on endothelial function since it has the ability to increase NO synthesis that, in vivo, plays an antioxidant function in the endothelium [18–20]. Animal models showed a relationship between prenatal and neonatal zinc deficiency and vascular dysfunction [21, 22] keeping in mind that zinc has antioxidant-like properties in activated endothelium cells [23]. Magnesium, an essential mineral for human health, plays a role in endothelium function and participates in vascular calcification [24]. Magnesium supplementation revealed a significant improvement of flow-mediated dilation and pulse wave velocity [25]. Recent findings showed the active role of selenium in endothelial function [24]. Specifically, selenium and seleno protein are associated with endothelial cytoprotection [24], having a role on endothelium activation biomarkers [26]. Among several functions, vitamin D is associated with endothelial dysfunction [27] showing anti-inflammatory effects through suppression of tumor necrosis factor-α and the release of interleukin-6 [28].

To the best of our knowledge, we did not find information regarding studies done in pediatrics, looking at endothelium dysfunction treatment. In light of these findings, using a double-blind randomized control study with a rigorous approach, we tested the effects on the endothelium of a combination of *Curcumin, resveratrol*, plus zinc, magnesium, selenium, and vitamin D in a cohort of pediatric subjects with obesity.

## Methods

### Participants selection

In this study, we recruited 48 children aged 6–17 years who were obese as defined by a body mass index (BMI) higher than the 95 percentile for age based on the Centers for Disease Control and Prevention standard [29]. Children with genetic syndromes or cardiovascular diseases were excluded from the study. The study was approved by the local Ethical Committee (OBELIX: code CE 5384, 2019). Informed written consent for study participation was collected from legal caregivers of each participant and from participants older than 10 years during the first visit. Participants were asked to attend three visits at 0, 3, and 6 months (±15 days).

### Randomization

This study was a double-blind randomized control study done with a rigorous approach. Using a computer-generated randomization schedule, study supplement and placebo were randomized (1:1) into 70 batches (each consisting of 6 packs containing 30 tablets a pack) and each was given a unique identification number. The coordinator of the study maintained the randomization list. Study physicians, other study personnel, and parents or legal guardians were blinded to the batches of medication and to the identification. Subjects who satisfied the inclusion for the study were assigned an identification number (linked to a batch) in sequential order. Since the randomization list was made before the batch assignment and later preserved in a closed envelope that made it unavailable for the entire duration of the study, neither study physicians nor patients could know subjects belonging to Placebo or Treatment group.

Subjects took one tablet per day orally starting day 1 after the visit and continuing for the 6-month duration of the study. A number of tablets not taken ≥2 tablets per month were considered not adherence to the study (drop out).

### Supplements characteristics

*Treatment tablet composition* (Auxilie® Immuplus, Envicon Medical, Verona, Italy): vitamin D3: 25.00 μg, folic acid: 90.00 μg, selenium: 55.00 μg magnesium: 300.00 mg, zinc: 7.00 mg, curcum (Meriva®): 100.00 mg, Polygonum dry extract: 20.,41 mg (of which resveratrol: 20.00 mg), Soy dry extract: 37.50 mg.

*Placebo tablet composition:* saccharose, fructose, aroma, anti-agglomerate agents: fatty acids, magnesium salts, silicium dioxide, colorant: riboflavin 5-sodium phosphate; sweetener: stevia glycoside, sucralose, neohesperidin DC.

Both tablets (treatment and placebo) were similar in form, color, and flavor.

### Anthropometric measurements and habits

Height (cm) and weight (kg) were measured for each child at every visit. BMI (kg/m^2^) was calculated as raw value and as *Z*-score for age. At the first visit, we conducted an oral interview with both parents and children and we collected information regarding sport/exercise practices and dietary habits.

### Endothelial function

Endothelial function was assessed using two methodologies: a post-occlusive release hyperemic test (PORH) and a heat provocation test (HPT). Subjects were laid on a bed with the upper extremity positioned at 45° and a cuff was placed around the medium third of the forearm in order to occlude the radial and ulnar arteries. The probe was positioned over the volar aspect of the hand at the first finger distal metacarpal surface and the hand was gently immobilized in order to minimize the occurrence of motion artefacts. All tests were performed with a laser Doppler sensor (Periflux 6000 System integrated with a thermostatic 457 probe, Perimed, Sweden).

#### Post-occlusive release hyperemia

Once cutaneous blood flow over the area became stable, basal values were recorded for 2 min and then the pressure within an inflatable cuff placed at the forearm and connected to a computer-controlled manometer was raised to 200 mm Hg for 3 min. Using a computer-controlled pressure release to allow for consistent deflation times, the cuff was rapidly deflated and the laser Doppler measured hyperemic responses over the next 2 min. Commercially available software (Perimed, Järfälla, Sweden) allowed for unbiased estimates of DF, hyperemic AUC and hyperemic AUC index.

#### Delta flow

The flow variation from resting flow to peak flow (maximal arterial flow achieved after abrupt cessation of occlusion) is called DF. DF was computed at baseline, after 3 months and after 6 months in each group. DF was compared between the two groups and its temporal trend was evaluated.

#### Hyperemic AUC and hyperemic AUC index

Hyperemic AUC is the difference between the area under the hyperemia zone and the area under the rest flow zone expressed in perfusion units multiplied by time. We also chose to compute an index named “hyperemic AUC index” in order to better reflect the real value of AUC in relation to the resting flow of each participant. AUC index = AUC/RF.

#### Heat provocation test

After the PORH test, we wait at least 2 min to proceed with the HPT in order to re-establish basal blood flow under the probe. The HPT consisted of recording resting blood flow in the forearm for 2 min, heating the forearm by raising the temperature of the probe to 44 °C and recording the hyperemic response induced by the heat expressed as a percentage difference in perfusion units above resting flow. This was defined as DF_HPT_.

### Statistical analysis

Qualitative variables were expressed as percentages and 95% confidence intervals (95% CI), and quantitative variables as means SD or medians and interquartile ranges depending on whether the variables were normally distributed. The Kolmogorov–Smirnov test was used to ascertain the normal distribution. Student’s *t* test and analysis of variance or Mann–Whitney test, Wilcoxon’s signed-rank test and Kruskal–Wallis test were used as appropriate to the data to compare distributions of DF, hyperemic AUC index and DF_HPT_ increase between Control and Treatment groups at fixed time intervals (0, 3 and 6 months) and to evaluate the trend of changes over time within each group. The association between each of these Periflux parameters at baseline (*T*0) and some relevant population characteristics such as BMI, gender, ethnicity, dietary fat intake, sport type and hours per week was tested using Pearson’s *r* correlation coefficient or Spearman’s correlation coefficient as appropriate. MedCalc Statistical Software version 17.6 (MedCalc Software bvba, Ostend, Belgium; http://www.medcalc.org) and GraphPad Prism version 6.00 for Mac (GraphPad Software, La Jolla California USA, http://www.graphpad.com) were used to perform the analyses and *α* was set at 0.05.

## Results

Forty-eight subjects were recruited and submitted to a questionnaire, medical examination, anthropometric evaluation and measurement of endothelial function. Twenty-one subjects dropped out. Nine subjects were not compliant with tablet prescription (two or more tablets not taken each month), whilst 12 patients did not attend 3- and 6-month follow-up due to personal reasons (mainly young subjects who found the 3 min cuff occlusion too annoying to tolerate).

Among the patients who completed the study, 16 patients took antioxidant supplementation and 11 took placebo. No one reported adverse effects.

The characteristics of all subjects at baseline are shown in Table 1. The characteristics and homogeneity of the subjects who completed the study are shown in Table 2. “Treatment group” and “Placebo group” were compared at baseline and were homogeneous with no significant difference in anthropometric and endothelial function parameters.

**Table 1:** Baseline Characterisitics of the study population.

	*N*	%
Patients enrolled	48	
Drop-outs	21	43.75
Age (years)	12.85 ± 3.04	
Gender (male)	25	52.08
Weight (kg)	76.90	[66.0–92.5]
BMI	30.89 ± 5.22	
BMI *Z*-score	0.61	[0.22–1.12]
Ethnicity		
Caucasic	41	85.42
North African	4	8.33
Moroccan	3	6.25
Sport		
Sport type score^a^	3	[2–3]
hours/week	3	[1–4]
Dietary fat intake		
Low	23	47.92
Medium	23	47.92
High	2	4.17
Drugs		
d-Vitamin	8	16.67
Methylphenidate	1	2.08
Montelukast	1	2.08
None	38	79.17

Data are expressed as number (*N*) and percentage or mean ± SD or median [IQR] as appropriate.

^a^Sport type = (dynamic score) × (static score).

**Table 2:** Characteristics of completers: Placebo vs Treatment groups.

	Control		Treated		*P* value
*N*	11		16		
Age (years)	11.4	[9.2–14.7]	12.8	[11.2–15.3]	NS
Gender (male)	7	63.6	9	56.3	NS
Weight (kg)	70.7	[65.0–113.0]	76.3	[66.9–90.0]	NS
BMI	30.4	[27.0–40.1]	30.6	[27.8–36.0]	NS
BMI *Z*-score	0.8	[0.1–1.7]	0.6	[0.2–0.9]	NS
Ethnicity					NS
Caucasic	7	63.6	13	81.3	NS
North African	3	27.3	1	6.3	NS
Moroccan	1	9.1	2	12.5	NS
Sport					NS
Sport type score^a^	3.0	[2.0–3.5]	2.5	[0.0–3.0]	NS
hours/week	2.5	[1.0–4.0]	2.0	[0.0–4.0]	NS
Dietary fat intake					NS
Low	6	54.5	7	43.8	NS
Medium	4	36.4	7	43.8	NS
High	1	9.1	2	12.5	NS
Periflux baseline data					NS
Resting flow	103.0	[42.0–176.0]	89.0	[47.5–137.3]	NS
Biological zero	8.0	[8.0–13.0]	9.0	[7.0–12.0]	NS
Peak flow	201.0	[146.0–274.0]	209.0	[159.5–232.3]	NS
Delta flow	79.0	[48.0–107.0]	117.0	[70.3–143.5]	NS
Hyperemic area (HA)	3015	[1745–4108]	4336	[2489–8194]	NS
HA index	27.9	[10.0–77.3]	49.4	[20.6–133.7]	NS
Pre-heat laser Doppler	122.0	[38.0–179.0]	84.0	[50.0–123.0]	NS
Post-heat laser Doppler	232.0	[132.0–278.0]	210.0	[187.3–278.0]	NS
Laser Doppler increase (%)	118.0	[20.0–219.5]	244.3	[71.9–397.1]	NS

Data are expressed as median [IQR] or number and percentage as appropriate.

*AUC* area under the curve, *HPT* heat provocation test, *NS* not significant.

^a^Sport type = (dynamic score) × (static score)

### Delta flow

**Fig. 1:**
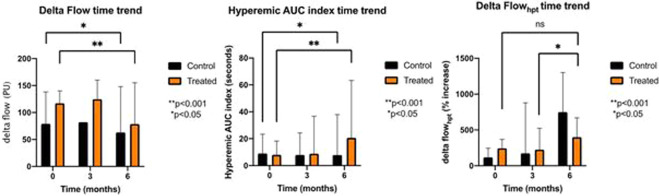
Time trend variation of Periflux data. Difference in the flow time trend between baseline, time-3-months, and time-6-months both in the placebo group and in the treatment group. *p < 0.05. **p < 0.001. ns not significant, PU perfusion unit, AUC area under the curve, HPT heat provocation test.

**Fig. 2:**
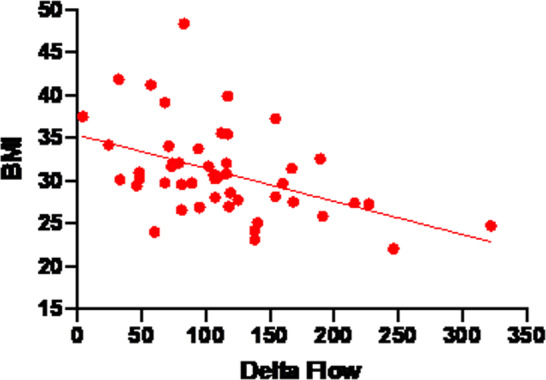
Correlation of delta flow and body mass index (BMI). This correlation increases with time and is stronger at time-6-months (*r* = 0.677).

“Treatment group” and “Placebo group” did not show differences in PORH DF at baseline and 3 months (Table 3). On the other hand, a significant difference was present at 6 months (78.5 [72.0–94.0] vs 63.0 [53.0–78.0], respectively, treatment and control, *p* < 0.001). Overall time trend of “Treatment group” and “Placebo group” was detected and compared with Kruskal–Wallis test, showing a significant difference in the flow time trend between baseline, 3 months, and 6 months both in the “Placebo group” (*p* < 0.05) and “Treatment group” (*p* < 0.001) (Fig. 1). Moreover, the comparison between the two groups was highly significant (*p* < 0.001). Regarding the correlation between anamnestic–anthropometric data and endothelial function parameters at baseline, the flow variation from resting flow to peak flow (DF) showed a significant correlation with BMI (Spearman’s *r* = 0.355, *p* < 0.05). The strength of this correlation increases with time; specifically, the correlation between BMI and DF was stronger at 6 months (*r* = 0.677) (Fig. 2 and Table 4).

**Table 3:** Periflux data of endothelial function.

	PORH												LD					
	RF (PU)		BZ (PU)		PF (PU)		DF		HA (PU*sec)		HA index		Pre-heat LD		Post-heat LD		LD increase (%)	
Basal																		
Control	103.0	[42.0–176.0]	8.0	[8.0–13.0]	201.0	[146.0–274.0]	79.0	[48.0–107.0]	3015	[1745–4108]	27.9	[10.0–77.3]	122.0	[38.0–179.0]	232.0	[132.0–278.0]	118.0	[20.0–219.5]
Treated	89.0	[47.5–137.3]	9.0	[7.0–12.0]	209.0	[159.5–232.3]	117.0	[70.3–143.5]	4336	[2489–8194]	49.4	[20.6–133.7]	84.0	[50.0–123.0]	210.0	[187.3–278.0]	244.3	[71.9–397.1]
3 months																		
Control	58.0	[51.0–125.0]	9.0	[7.0–13.0]	186.0	[96– 222.0]	99.0	[51.0–148.0]	4202	[1283–6140]	49.7	[18.2–87.0]	86.0	[32.0–136.0]	193.0	[167.0–264.0]	174.1*	[32.0–807.7]
Treated	74.5	[53.8–104.3]	9.5	[6.5–11.5]	201.5	[164.8–224.0]	124.5	[71.3–157.0]	4114	[2328–8390]	52.2	[25.8–127.9]	77.0	[57.3–89.8]	237.0	[212.5–310.5]	226.1*	[133.8–518.9]
6 months																		
Control	41.0	[21.0–70.0]	7.0	[6.0–15.0]	116.0	[82.0–189.0]	63.0**	[53.0–78.0]	2434	[1766–3588]	78.2	[16.1–141.5]	32.0	[15.0–41.0]	164.0	[154.0–183.0]	749.0*	[293.0–1196.0]
Treated	43.5	[35.8–51.8]	7.0	[5.3–14.5]	119.0	[113.0–176.0]	78.5**	[72.0–94.0]	2814	[1674–4350]	72.0	[32.5–114.5]	40.5	[33.3–56.8]	191.5	[163.0–211.8]	400.0*	[232.5–415.8]

*PORH* post-occlusive reactive hyperemia, *LD* laser Doppler, *RF* resting flow, *BZ* biological zero, *PF* peak flow, *HA* hyperemic area, *PU* perfusion unit.

DF = peak flow − resting flow.

Hyperemic area index = hyperemic area/resting flow.

*p < 0.05.

**p < 0.001.

**Table 4:** Correlation coefficients of Periflux parameters with anthropometric data.

	BMI		Gender (male)		Ethnicity		Sport type score		Dietary fat intake		Sport (h/week)	
Delta flow	−0.457**	[−0.661; −0.191]	0.015	[−0.278; 0.306]	0.156	[−0.143; 0.428]	−0.079	[−0.363; 0.218]	−0.116	[−0.407; 0.196]	0.099	[−0.209; 0.389]
HA index	−0.108	[−0.388; 0.190]	0.348*	[0.062; 0.581]	−0.084	[−0.367; 0.214]	−0.079	[−0.363; 0.218]	0.105	[−0.207; 0.397]	0.114	[−0.195; 0.402]
LD increase (%)	−0.213	[−0.476; 0.084]	0.133	[−0.166; 0.409]	0.057	[−0.239; 0.344]	−0.043	[−0.331; 0.252]	−0.339*	[−0.583; −0.037]	−0.147	[−0.430; 0.162]
Delta flow (6 months)	−0.677**	[0.237; 0.887]										

Data are expressed as Spearman’s *r* coefficient and related 95% CI.

*PU* perfusion unit, *95% CI* 95% confidence interval, *HA*: hyperemic area, *LD*: laser doppler.

Delta flow = peak flow − resting flow.

Hyperemic area index = hyperemic area/resting flow.

Ethnicity: Caucasic > North African > Moroccan.

*p < 0.05.

**p < 0.001.

### Hyperemic AUC index

“Treatment group” and “Placebo group” did not show mutual differences in “hyperemic AUC index” at 3 and 6 months (Table 3). An overall time trend of “Treatment group” and “Placebo group” was detected and compared with Kruskal–Wallis test, showing a significant difference in the Hyperemic AUC index both in the “Placebo group” (*p* < 0.05) and “Treatment group” (*p* < 0.001) (Fig. 1). In addition, the comparison between the time trends of the two groups was very significant (*p* < 0.001). About the relationship between anamnestic and anthropometric data and endothelial function parameters at baseline, the hyperemic AUC index showed a significant correlation with the male gender (Spearman’s *r* = 0.348, *p* < 0.05) (Table 4).

### Delta flow heat provocation test

“Treatment group” and “Placebo group” showed a significant difference between groups in DF_HPT_ when comparing the results of the “HPT” at 3 months and 6 months (*p* < 0.05) (Table 3). The overall time trend of the “Treatment group” and “Placebo group” was detected and compared with Kruskal–Wallis test. In the “Placebo group,” there was no significant difference in the DF_HPT_ analysis between baseline, 3 months, and 6 months. In the “Treatment group,” DF_HPT_ analysis did not show a significant difference between baseline and 3 months. However, there was a significant difference between 3 and 6 months (*p* < 0.05) in this group (Fig. 1). Regarding the association between anamnestic and anthropometric data and endothelial function parameters at baseline, DF_HPT_ did not show a significant correlation with anamnestic and anthropometric parameters (Table 4).

## Discussion

The results of the study illustrate the correlation between obesity status and endothelial dysfunction in children, showing that cardiovascular damages begin early in life. Although the instrument and the methodology in use in this study are considerably new, we used the most validated parameters such as DF, hyperemic area under the curve and DF_HPT_, to estimate endothelial function or dysfunction in adults [30]. Previous studies showed that endothelial dysfunction was associated with adiposity in obese children as well [6, 31, 32].

Our results showed that DF improved significantly (*p* < 0.001) in the Treatment group when compared with the Control group. It should be noted that all the enrolled subjects, both treatment and controls, have had some benefits in their endothelial function over time, perhaps due to the fact that some educational advice was given during the medical assessment. However, in the Treatment group, this benefit was constantly and significantly higher (*p* < 0.001), implying a role of the supplements in promoting a higher degree of improvement. The correlation between basal DF and basal BMI shown in Fig. 1 is negative, indicating that DF decreases while BMI increases, once again, underlining the treatment effect in enhancing endothelial performances despite an increase in the BMI.

Although we did not find any correlation with dietary fat content, type, and hours spent on physical activities, sports, or ethnicity, we may speculate that a larger sample size and/or different ethnicity could show significant results. It is important to recognize that our population was mostly Caucasian. Regarding physical activity, previous studies showed improvement of arterial stiffness, reduction of abdominal fat, increases cardiorespiratory fitness, and delayed arterial wall remodeling in prepubertal obese children [31].

We did not find any significant correlation between groups in the area of hyperemia, showing a similar trend of time variation in both groups. The influence of advice by the physician in promoting changes in both groups cannot be excluded. However, the greater changes in the treated group in comparison with the control group suggests a treatment effect of the supplements in modifying the “hyperemic area under the curve.” Again, a larger sample size or an even longer observational/treatment period might influence future results.

Regarding gender, males showed a wider range of hyperemia in comparison with females and despite the small sample size, North African subjects showed a higher range of hyperemia, followed by Italo-Moroccan and Caucasians. In contrast with our results, Mueller et al. [33] found that in females endothelium function was lower than in men. However, the cohort of adolescents in the study of Mueller et al. [33] was mainly healthy. Adult men usually develop cardiovascular diseases at a younger age and have a higher propensity to develop coronary heart diseases than women [34].

Looking at the “DH provocation test,” significant changes between the treated group and control group were found after 3 months of treatment (*p* < 0.05), giving us the impression that the combination of *Curcumin* and *resveratrol* plus zinc, magnesium, selenium, and vitamin D require some time to influence endothelium function per se.

Regarding the relationship between endothelial function and BMI, a recent systematic review and meta-analysis in adults showed that diet improved endothelium function independently from BMI [35], but we did not find similar results in children using the same approach and measurements.

In summary, *Curcumin*, *resveratrol*, zinc, magnesium, selenium, and vitamin D appear to be promising in enhancing endothelial function by improvement of both DF in the PORH test and DF in the heat provocation test, although it seems the combination effectively acts at least after 3 months since the therapy started.

The primary mechanism of action of polyphenols was originally thought to lie in their direct antioxidant effects. However, a number of other possible biochemical and molecular mechanisms have been identified, including multifarious effects within intra- and intercellular signaling pathways that govern antioxidative properties like nuclear factor E2-related factor 2 and inflammation pathways, e.g., nuclear factor-κB and thus modulating the synthesis of inflammatory mediators including cytokines tumor necrosis factor-α, interleukin-1β (IL-1β), and IL-6 [36–38]. Furthermore, *Curcumin* and *resveratrol* have been demonstrated to exert epigenetic regulatory roles including the inhibition of DNA methyltransferases, regulation of histone modifications via the regulation of histone acetyltransferases and histone deacetylases, regulation of microRNAs, and action as a DNA-binding agent [38, 39]. The accumulation of visceral fat in obesity is associated with a state of chronic oxidative stress and excessive production of proinflammatory adipokines, which contributes to a low-grade chronic inflammation state that can be attenuated with magnesium [40], zinc [41], and selenium supplementation as recognized antioxidant trace elements [42]. The effects we observed have biological plausibility. Daily vegetable consumption was associated with more favorable arterial function [43] and the same was observed increasing the polyphenol content of the diet via consumption of fruit and vegetables [44], with a dose-dependent effect [45]. Red wine consumption has been shown to positively influence processes involved in vascular dysfunction [46] and *resveratrol*, one of the main compounds of the tablets used in our study, is the major polyphenol in wine. A systematic review and meta-analysis of randomized controlled trials clearly documented that *resveratrol* [47] or *curcumin* [48] intervention significantly increased flow-mediated dilatation. Several studies documented that a high intake of dietary polyphenols inhibit endothelial dysfunction and induce vascular endothelium-dependent vascular relaxation viz. redox regulation and NO production [47] and may have a preventive effect against cardiovascular diseases [49, 50]. Moreover, vitamin D insufficiency is associated with increased arterial stiffness and endothelial dysfunction [51], and vitamin D levels are inversely associated with increased arterial stiffness in a normative aging population, irrespective of the traditional risk factor burden [52]. Its supplementation improved NO-dependent arteriolar vasodilation in obese adults [53] and is associated with decreased vascular dysfunction in patients with chronic kidney disease [54]. Hard water consumption seems to be protective against cardiovascular diseases particularly in relation to its magnesium content [55] and a meta-analysis suggest that oral Mg supplementation may improve endothelial function when conducted at least for 6 months and in unhealthy, overweight or older individuals [56] as observed in our studied children. In animal models, zinc deficiency is associated with reduced vasodilator response [21], and in humans, zinc supplementation alleviates diabetic endothelial dysfunction [22]. Zinc is a protective and critical nutrient for the maintenance of endothelial integrity [23] through attenuation of tumor necrosis factor-mediated activation of upregulation of inflammatory cytokines in endothelial cells [57]. In rats, it has been documented that hypercholesterolemia, a condition commonly found in obese subjects, promotes endothelial dysfunction in the presence of selenium deficiency [58] and many studies in humans indicate an association between selenium deficiency and increased risk of morbidity and mortality [24]. Selenium supplementation has been shown to significantly reduce the risk for cardiovascular mortality in patients with diabetes, hypertension, and ischemic heart disease [59].

According to the study results, looking at the day-by-day clinical work together with regular physical activity, an increased consumption of the correct diet containing nutritive and non-nutritive compounds may contribute to the improvement of the quality of life by delaying the development of endothelial dysfunction in pediatric subjects with obesity.

Despite intriguing findings, our study has some limitations. The high drop-out level we had in our study population is in line with the low adherence of obese children to health-behavior recommendations [60] and this fact may also be responsible for the partial effect we observed with the nutraceutical supplementation. Moreover, the potential effect of growth on our subjects has not been taken into consideration in the study.

It is well known that the laser Doppler technique used to evaluate the endothelial function and ultimately cardiovascular risk in adults is used only in research and no clinical studies can be found. We did not find any guideline or protocol to diagnose endothelial dysfunction using this technique. Another important point that needs to be taken into consideration is the time during the day of the vascular assessment. We know that fasting and “circadiam rhythm” may influence results per se and we did not have the possibility to take measurements at the same time of the day, although we tried very hard to standardize the time of measurements.

Treatment of obesity is always an issue and since there is a high prevalence of inflammation in obese subjects leading to several problems including endothelial dysfunction and related cardiovascular complications [61], substances with antioxidant, anti-inflammatory, angiogenic, and platelet aggregation properties such as resveratrol and curcumin, zinc, selenium, magnesium, folic acid, and vitamin D [16, 61, 62] may have protective effects to improve endothelial dysfunction, thus lowering the risk of developing cardiovascular diseases [63]. In conclusion, it is possible to speculate that multicomponent supplementation with minerals, vitamin D, and functional food-derived factor that resembles but not a substitute for a healthy diet may help to improve vascular dysfunction in obese children [8].
