# Correction: Supportive treatment of vascular dysfunction in pediatric subjects with obesity: the OBELIX study

**DOI:** 10.1038/s41387-022-00183-6

**Published:** 2022-01-20

**Authors:** Luca Pecoraro, Thomas Zoller, Richard L. Atkinson, Fulvio Nisi, Franco Antoniazzi, Paolo Cavarzere, Giorgio Piacentini, Angelo Pietrobelli

**Affiliations:** 1 Department of Medicine, University of Verona, Verona, Italy; 2 Paediatric Clinic, ASST Mantua, Mantua, Italy; 3 Pediatric Unit, Department of Surgical Sciences, Dentistry, Gynecology and Pediatrics, University of Verona, Verona, Italy; 4 Department of Medicine, Virginia Commonwealth University, Richmond, VA USA; 5 Humanitas Clinical and Research Center—IRCCS, Rozzano, MI Italy; 6 Pennington Biomedical Research Center, Baton Rouge, LA USA

**Keywords:** Cardiovascular diseases, Obesity

Correction to: *Nutrition and Diabetes* 2022;12:1–8 10.1038/s41387-021-00180-1, published online 10 January 2022

The original version of this article unfortunately contained a mistake. Due to a typesetting error, the author names were interchanged. We apologize for the error. The original article has been corrected.

